# 

**Published:** 1915-03

**Authors:** 


					﻿PUBLISHED MONTHLY.	ATI-ANTA	SUBSCRIPTION $1.00
JOURNAL-RECQRD
OF MEDICINE
c I < p - p. ,
Atlanta Medical and Surgical Journal, Establlshed'1$55. yp-j » Vd'ir
and Southern Medical Record, Established 1870. yutSl IVUI
Vol. LXI.	Atlanta, Ga., March, 1915.	No. 12^
				

